# First record of microplastic contamination in adult endemic amazonian anuran species

**DOI:** 10.1038/s41598-025-86434-9

**Published:** 2025-01-18

**Authors:** Maria Luiza Cunha e Souza-Ferreira, Adrian José Oliveira dos Reis, Erikson Bruno Loseiro Ferreira, Jessica Dipold, Anderson Z. Freitas, Niklaus U. Wetter, Verônica Regina Lobato de Oliveira-Bahia, Thiago Bernardi Vieira

**Affiliations:** 1 Multidisciplinary Laboratory of Animal Morphophysiology, Federal University of Pará, Belém, Pará 66075- 110 Brazil; 2 Laboratory of Conservation Biogeography and Macroecology, Federal University of Pará, Belém, Pará 66075-110 Brazil; 3 Institute of Energy and Nuclear Research, IPEN-CNEN, São Paulo, SP 05508-000 Brazil; 4 Ecology Laboratory, Federal University of Pará - Altamira campus, Altamira, Pará 68372-040 Brazil

**Keywords:** Herpetofauna, Plastic, Polymers, Frogs, Microhabitat, Ecology, Environmental sciences

## Abstract

The microplastics (MPs), due to their high dispersion and bioaccumulation rates, have been identified in various animal groups, such as anuran amphibians during both larval and adult stages. However, current studies on adult anuran amphibians focus on assessing only one exposure route, the digestive system, while other routes remain underestimated. Therefore, this present study aimed to evaluate the degree of contamination in the digestive, respiratory, and integumentary systems, in situ, of two endemic Amazonian adult anuran species (*Physalaemus ephippifer* and *Boana multifasciata*). From this, we identified and characterized microplastic particles for each exposure route, assessed the effects of morphometric measures on the total MP contamination level and in the gastrointestinal tract (GIT). Additionally, we determined different contamination indices and how they vary according to species and systems. Based on our data, the digestive and integumentary exposure routes showed the highest contamination levels for both species. Additionally, variations in MP contamination levels indicated that *P. ephippifer* had a higher level of MP contamination. Thus, this study provides the first evidence of microplastic exposure through respiratory and integumentary routes in adult anurans in situ, and it is the first to identify MP contamination in terrestrial biomonitors in the Amazon.

## Introduction

Plastic waste contamination is a global issue, intensified by the presence of smaller particles that disperse more readily through various transport pathways, including airborne^1^ and aquatic routes^2^. Plastics are anthropogenic, fossil-based synthetic polymers widely utilized in industry due to their high durability and resistance^3^. Plastic particles smaller than 5 mm and larger than 1 μm, known as microplastics (MPs), originate either as primary MPs, intentionally manufactured at this size, or as secondary MPs, formed through the fragmentation of macroplastics (> 5 mm) via environmental weathering, photodegradation, or chemical and biological processes. Microplastics can exhibit diverse morphologies, including fibers, fragments, and granules, with their size and form playing a critical role in their environmental behavior and impacts on biota^4,5^.

Contamination by microplastics (MPs) has been evidenced in various zoological groups, including fish^6,7^, mammals^8^ ,reptiles^9^, and anurans^10–12^. Among these, anurans (amphibians belonging to the class Amphibia) stand out due to their biodiversity loss linked to anthropogenic activities, such as habitat loss and fragmentation, exposure to toxic metals and pathogens, and the introduction of exotic species^13,14^. Anuran amphibians provide essential ecosystem services, such as biological control and bioturbation^15^, and serve as important food sources for various species (e.g., lizards, snakes, and birds)^16^ and are considered excellent models for ecotoxicological studies, such as bioindicators and biomonitors of aquatic and terrestrial environments, given the dual life cycle of many species^17,18^. In their larval phase, exposure to MPs leads to the accumulation of these particles in tissues, particularly the liver and intestines, with contamination levels influenced by exposure duration and environmental conditions^19,20^. In adults, studies primarily focus on MP accumulation in the gastrointestinal tract (GIT)^11^, while studies with tadpoles have demonstrated that exposure can occur in two ways: incidental^21^ or trophic transfer^20^. In tadpoles, effects such as tissue damage and intestinal obstruction have been observed, which may lead to alterations in growth, development, behavior, and survival^17,20,22^.

In addition to the digestive route, the MPs can be exposed through other pathways, such as dermal^12^ and inhalation^8^. However, knowledge about other exposure routes to MPs in adult anurans remains limited, primarily the inhalation route^17^. Exposure through respiratory pathways mainly occurs due to atmospheric transport^23^, resulting in various damages to organisms, such as epithelial barrier dysfunction, cytotoxicity, and synergistic effects with allergens. Additionally, the tissue accumulation of these particles in the respiratory epithelium can cause chronic inflammation, granuloma, and fibrosis^24,25^. However, the dermal contamination pathway in adult anurans has only been identified recently^12^. Despite this discovery, it remains largely underestimated, and its associated effects and mechanisms are still poorly understood. In humans, it has been observed that MPs exposed through the dermal route can penetrate the skin barrier, potentially causing damage to the liver and kidneys^26^. Effects on the functional biology of primary skin cells have also been observed in vitro assays^27^, as well as negative impacts on the maintenance of bodily fluid homeostasis in fish^28^.

Furthermore, species-specific habits can influence MP contamination levels in the organism^29^. Anuran habits can be classified into five classes: semi-aquatic (predominantly in the aquatic environment or near water), arboreal (above ground in trees or shrubs), fossorial (predominantly in leaf litter or burrows), torrential (associated with rocky and fast-flowing streams), and terrestrial (linked to the ground)^30^. Therefore, variations in anuran habits influence the degree of exposure to MPs^29^, both due to intrinsic characteristics of microhabitat use^30,31^ and the transport characteristics of MPs between environmental compartments^32^.

Thus, our objective is to assess the degree of contamination by microplastics in the digestive, respiratory, and integumentary systems in two endemic Amazonian adult anuran species with in situ exposure. For this purpose, we selected two model species: (i) Steindachner’s dwarf frog *Physalaemus ephippifer* (Steindachner, 1864), a species belonging to the *Physalaemus cuvieri* species complex, from the Leptodactylidae family, with nocturnal and terrestrial habits, usually found near temporary ponds and shallow lakes^33,34^ ; and (ii) Many-banded Treefrog *Boana multifasciata* (Günther, 1859) belonging to the Hylidae family, widely distributed in the Amazon, with nocturnal and arboreal habits (shrubby habit)^35^. Both species predominantly occur in the forest edge and clearing area, and they have an omnivorous diet, in addition to being easily identifiable, collected, transported, and widely distributed, and being abundant. From this, we aim to characterize the composition of shape and color of MPs in the two model species, identify the relationship between morphometric measures and the level of total MP contamination and in the GIT, and check the variation of MP contamination indices between the two species and according to different exposure routes.

## Results

### Characterization of microplastics

**Fig. 1:**
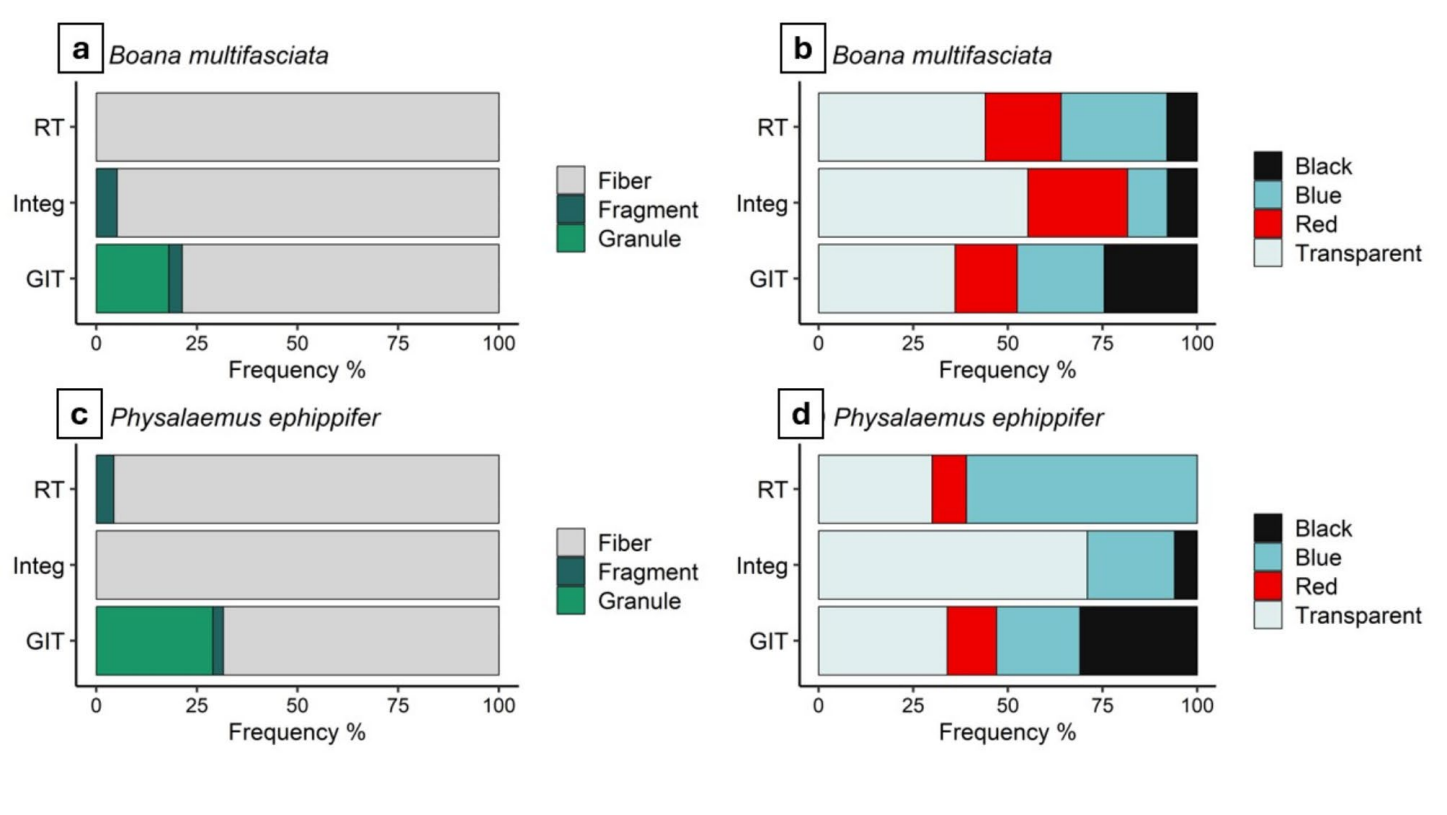
Composition of shape and color of microplastics (MPs) found in the respiratory tract (RT), gastrointestinal tract (GIT), and integument (Integ) in two species of anurans: *Boana multifasciata* and *Physalaemus ephippifer*. (**a**) Percentage frequency of MP shapes for *Boana multifasciata*. (**b**) Percentage frequency of MP colors for *Boana multifasciata*. (**c**) Percentage frequency of MP shapes for *Physalaemus ephippifer*. (**d**) Percentage frequency of MP colors for *Physalaemus ephippifer*.

**Fig. 2:**
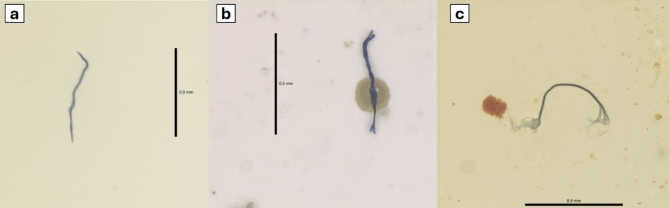
Photomicrograph (120x) of microplastic fibers found in different exposure pathways. (**a**) Blue microplastic fiber on the integument. (**b**) Blue microplastic fiber in the respiratory tract. (**c**) Blue microplastic fiber in the gastrointestinal tract.

We identified 237 MP particles, of which 92 (38.82%) were in *P. ephippifer*, and 145 (61.18%) in *B. multifasciata .* For the species *P. ephippifer*, 20 (66.66%) of the sampled individuals exhibited the presence of MP, while for *B. multifasciata*, 27 (90%) of the individuals had MP in at least one of the analyzed pathways (Table 1). In integument (Integ), we identified 90 MPs, of which 33 (36.66%) were in *P. ephippifer* and 57 (63.33%) in *B. multifasciata*. In respiratory tract (RT), 46 MPs were identified, of which 22 (47.82%) were in *P. ephippifer* and 24 (52.17%) in *B. multifasciata*. In gastrointestinal tract (GIT), the highest concentration of microplastic particles was identified, totaling 101 records, with 37 (36.93%) in *P. ephippifer* and 64 (63.36%) in *B. multifasciata*. The predominant form of MP was the fiber, with a total of 190 (80.16%) records (Fig. 1a, c). The predominant color was transparent, totaling 94 (39.66%) records, followed by blue with 53 (22.36%) MP records (Fig. 1b, d). Regarding the variation in the size of MPs according to the exposure pathway, there was no significant difference (X^2^ = 1.188, df = 2, p-value = 0.552), with an average of 2.36 + 1.77 mm in the GIT, 2.43 + 1.43 mm in TR, and 2.63 + 1.42 mm in Integ (Fig. 2).

**Table 1:** Microplastic (MP) contamination levels based on the number of contaminated individuals and percentage in the species *Boana multifasciata* and *Physalaemus ephippifer*, separately.

	Individuals with MP (%)	MP index Mean + SD			
		MP/ind	MP/g*	MP/SVL	MP GIT / mass GIT
*B. multifasciata*	27 (90%)	4.83 + 2.26	0.71 + 0.48	0.09 + 0.06	0.01 + 0.01
*P. ephippifer*	20 (66.66%)	3.53 + 3.52	2.17 + 2.27	0.13 + 0.13	0.016 + 0.02

Mean values and standard deviations for contamination indices for each species: total MP index (MP/ind.), MP index weighted by mass (MP/g), MP index weighted by SVL (MP/SVL), MP index in the gastrointestinal tract (MP GIT/mass GIT). MP: microplastic. SD: standard deviation. SVL: snout-vent length. GIT: gastrointestinal tract. (*) index showed statistical difference for mean analysis (PERMANOVA (*P* < 0.001)).

**Fig. 3:**
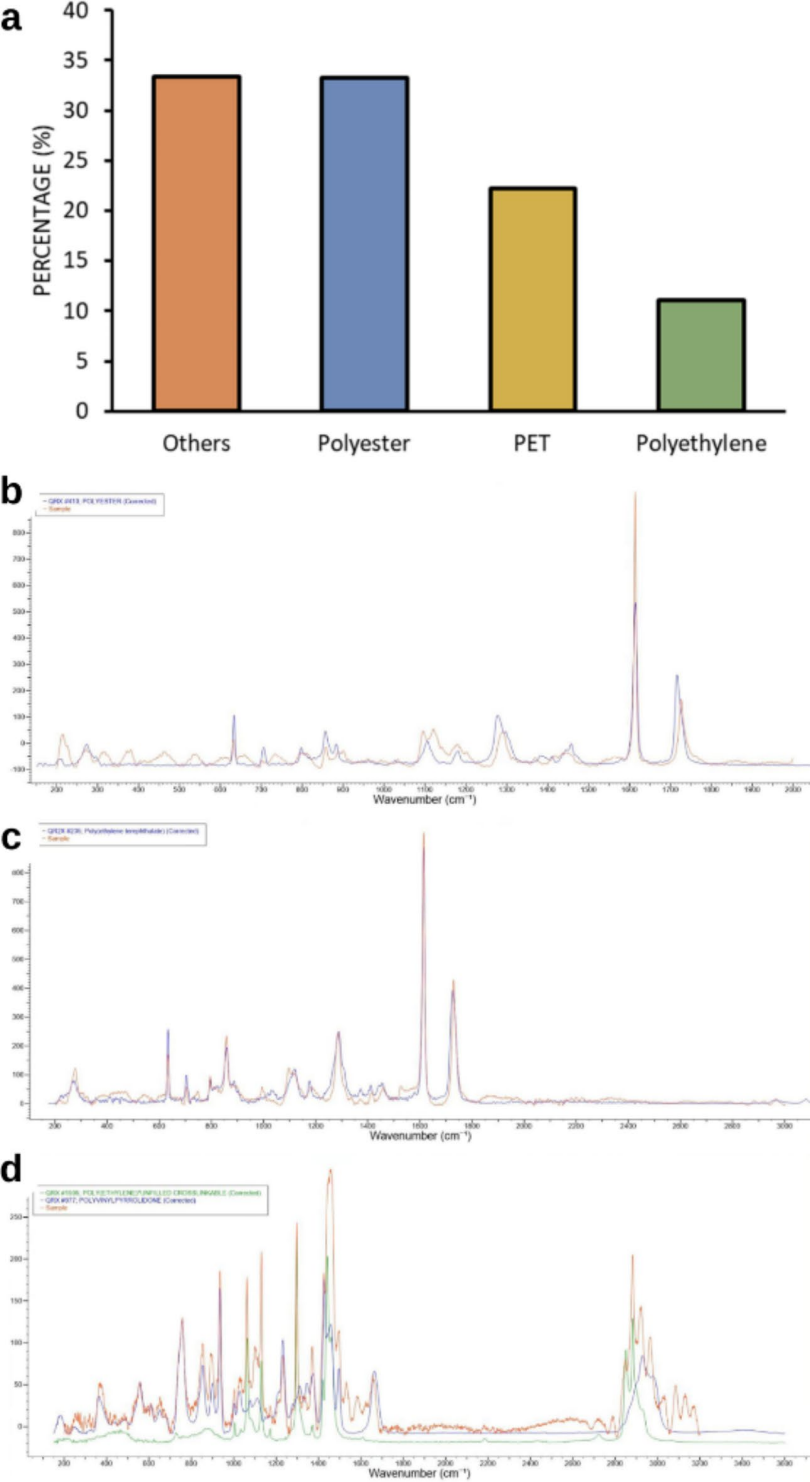
Chemical composition of polymers analyzed by Raman spectroscopy. (**a**) Percentage of materials detected in both species. (**b**–**d**) Representative Raman spectroscopy of polystyrene, PET, and polyester, respectively.

For the validation and characterization analysis of the samples, 78.1% of the samples had a conclusive spectral analysis. Among these, 33.3% were polyester (PS), 22.2% polyethylene terephthalate (PET), and 11.1% polyethylene (PE), identified in both species (Fig. 3). Additionally, P-(aryletherketone) and Poly(butene-1-sulfone) were identified once each. Among the non-plastic samples, the majority were identified as textile materials (75%), whether by their cellulose spectrum, lyocell (which is a synthetic fabric), or Indigo, which is a dye mainly used for denim. The other samples identified as non-plastic consisted of other dye colors and organic matter.

### Contamination indices among species

From the obtained morphometric measurements, for *P. ephippifer*, the mean total mass was 1.80 + 0.41 g, and the mean SVL (snout-vent length) was 26.75 + 2.18 mm, and for *B. multifasciata*, the mean total mass was 6.72 + 0.59 g, and the mean SVL was 48.70 + 2.03 mm. In the morphometric data of the GIT in *P. ephippifer*, the mean mass obtained was 109.61 + 79.36 mg, and the mean length was 67.12 + 19.69 mm, and for *B. multifasciata*, the mean mass was 320.93 + 157.07 mg, and the mean length was 103.89 + 13.39 mm. When analyzing the morphometric data of total mass and SVL, we did not observe variation related to the total contamination level by MP in both species (Table 2). On the other hand, in *B. multifasciata*, the variation obtained in MP contamination in the GIT showed a negative relationship with the mass and length of the GIT, where higher mass and greater GIT length reflected a lower level of contamination by MP in the GIT. For *P. ephippifer*, the observed relationship was opposite, with the relationship observed based on the mass of the GIT only, and this was positive (Table 2).

**Table 2:** Summary of the results obtained in the generalized Linear Model (GLM) when evaluating the effect of morphometric measures on the total microplastic (MP) contamination level and in the gastrointestinal tract (GIT) for *Boana multifasciata* and *Physalaemus ephippifer*, separately.

Variable	Boana multifasciata					Physalaemus ephippifer			
	Estimate	SE	Z	*p*		Estimated	SE	Z	*p*
MP total									
(Intercept)	-1.354	2.277	-0.595	0.552		**3.039**	**1.234**	**2.464**	**< 0.050**
Total mass	0.046	0.048	0.957	0.338		-0.026	0.047	-0.554	0.579
SVL	0.119	0.151	0.792	0.428		-0.437	0.284	-1.538	0.124
MP in GIT									
(Intercept)	**4.219**	**0.940**	**4.491**	**< 0.001**		0.128	0.520	0.246	0.805
GIT mass	**-0.003**	**0.001**	**-2.673**	**< 0.01**		**0.005**	**0.002**	**2.897**	**< 0.010**
GIT length	**-0.027**	**0.010**	**-2.395**	**< 0.05**		-0.007	0.008	-0.847	0.396

Predictor variables for the relationship with total MP: total mass and SVL (snout-vent length). Predictor variables for the relationship with MP in the GIT: mass and length of the GIT. MP: microplastic. GIT: gastrointestinal tract. Estimated: predicted effect of predictor variables on the response variable. SE: standard error. Z: standardized coefficient estimate for each predictor variable, indicating its relative influence on the model. Values in bold were statistically significant (*p* < 0.05).

**Fig. 4:**
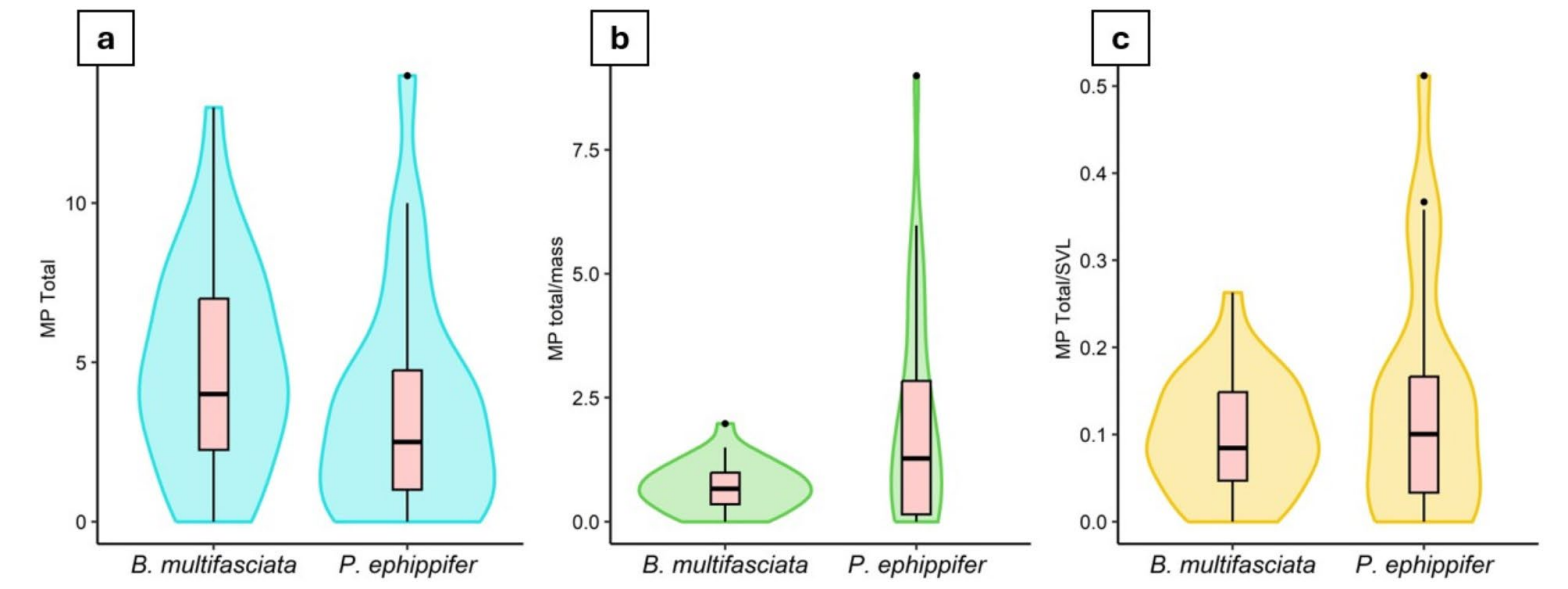
Microplastic contamination indices in *Boana multifasciata* and *Physalaemus ephippifer.* (**a**) Total microplastic contamination index per individual. (**b**) Microplastic contamination index weighted by mass. (**c**) Microplastic contamination index weighted by SVL. See Table 2 for statistical results.

We did not observe a difference in the total MP contamination index between species, considering the means (PERMANOVA (F_1,56_ = 2.080, *P* = 0.189), and variations in contamination between species (PERMDISP (F_1,56_ = 0.006, *P* = 0.995) (Fig. 4a). However, we observed a difference in the MP contamination index total weighted by total mass, both for the means (PERMANOVA (F_1,56_ = 10.422, *P* < 0.001), and for the variations (PERMDISP (F_1,56_ = 15.434, *P* < 0.001), with a higher mean and variation observed in the *P. ephippifer* species (Fig. 4b). In the MP contamination index weighted by SVL, we did not observe differences considering the means (PERMANOVA (F_1,56_ = 1.735, *P* = 0.204), however, the *P. ephippifer* species presented higher variance (PERMDISP (F_1,56_ = 6.635, *P* < 0.01) (Fig. 4c). For the contamination index in the GIT, there was no difference between species, neither for the means (PERMANOVA (F_1,56_ = 1.385, *P* = 0.260)), nor for the variances (PERMDISP (F_1,56_ = 1.610, *P* = 0.222)). The means and standard deviations of the contamination indices for each species are available in Table 1.

**Fig. 5:**
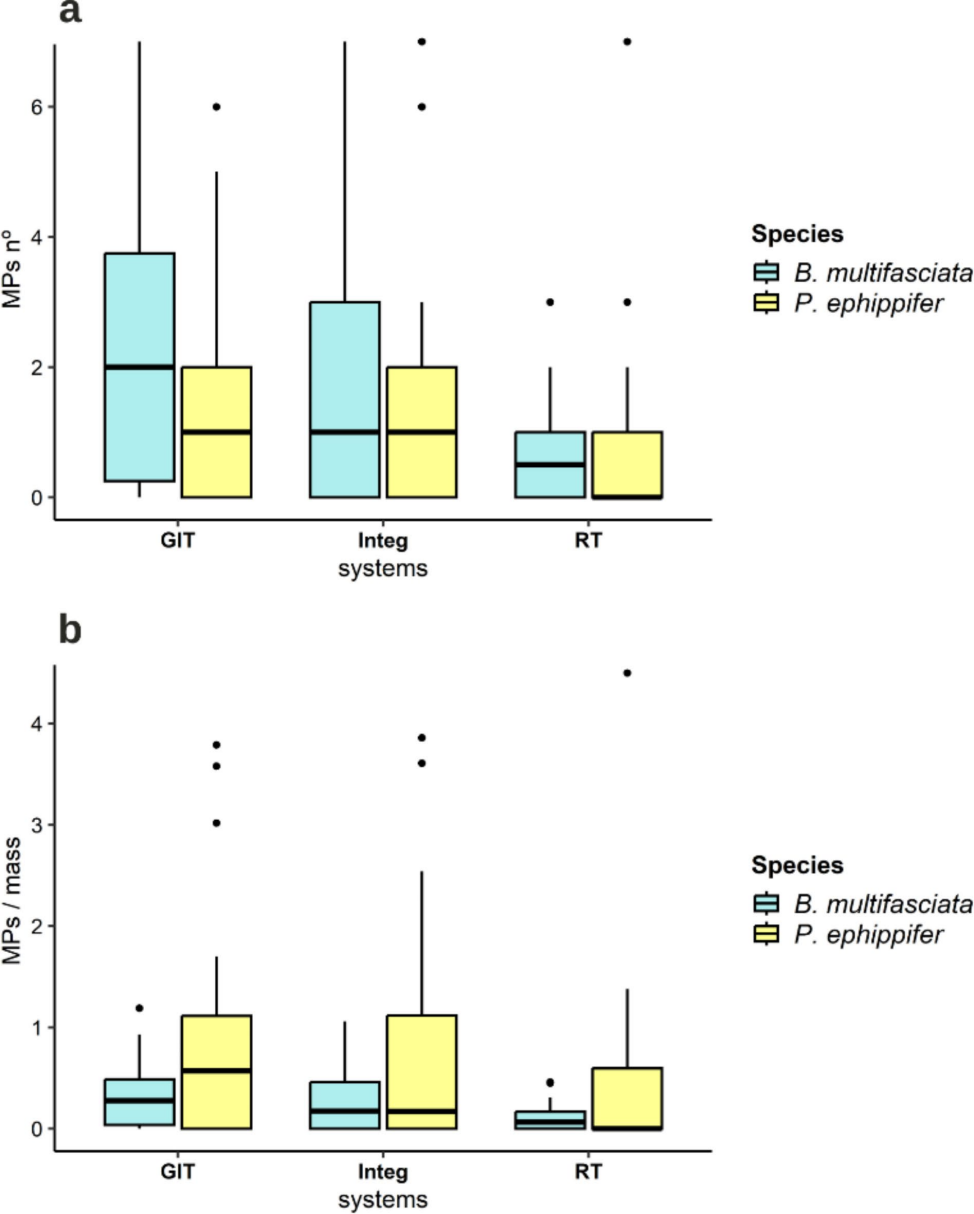
Contamination levels in *Boana multifasciata* and *Physalaemus ephippifer* according to each contamination pathway (systems): Integument (Integ), Gastrointestinal Tract (GIT), and Respiratory Tract (RT). (**a**) Mean absolute MP count for each system per species. (**b**) MP count weighted by total mass for each system per species.

When checking the variation in MP contamination for each system and species considering the absolute MP values, the observed difference in means was due to the variation between systems within each species, with no difference in terms of species (Table 3). While the variability of the samples did not differ significantly between groups (PERMDISP (F_5,159_ = 1.859, *P* = 0.097)). For this metric, it is possible to observe higher levels of contamination in the GIT and Integ for both species (Fig. 5a), with the difference in multiple variations observed between *B. multifasciata* RT and *P. ephippifer* GIT (*P* < 0.05). When evaluating the level of contamination by MP weighted by total mass for each system and species, the variation between the means obtained was due to the species (Table 3). When weighing the levels of contamination by MP, the species *P. ephippifer* begins to show higher contamination by MP in all evaluated systems (Fig. 5b), with a difference in variability between groups, which was greater in *P. ephippifer* (PERMDISP (F_5,159_ = 5.153, *P* < 0.01).

**Table 3:** Results of PERMANOVA concerning the analysis of MP, considering the species (*Boana multifasciata* and *Physalaemus ephippifer*) and the systems (integument, respiratory tract, and gastrointestinal tract) as predictor variables.

Variables	Absolute values of MP				MP weighted by mass			
	df	*R* ^2^	F*	*p*	df	*R* ^2^	F*	*p*
Specie	1	0.014	2.585	0.101	**1**	**0.094**	**17.013**	**< 0.001**
System	**2**	**0.057**	**5.022**	**< 0.001**	2	0.023	2.162	0.105
Specie x system	2	0.009	0.799	0.462	2	0.002	0.229	0.806
Residuals	175	0.919	-	**-**	175	0.879	-	-
Total	180	1.000	-	-	180	1.000	-	-

Results obtained from the absolute values of MP contamination for each system and values of MP weighted by total mass for each system, analyzed separately. Df: degrees of freedom. R^2^: correlation coefficient. F*: Pseud Fisher F. values in bold showed significant difference for mean analysis (*p* < 0.05).

## Discussion

Having assessed microplastic (MP) contamination in the gastrointestinal tract (GIT) and integument, this constitutes the initial report of MP presence in the respiratory tract of adult anurans in situ. In this context, we observed the presence of plastic contaminants in neotropical anuran amphibians, thereby supporting the vulnerability of this group to environmental exposure to MP^10–12,36^. Based on our analyses, we observed that microplastic (MP) contamination in anurans may reflect the environmental contamination level with less influence from body morphometric variables. This was evident as morphometric variables such as mass and SVL (snout-vent length) showed no significant effect on total MP contamination in both species. When comparing contamination indices between the two species, a higher level of total MP was observed in the *B. multifasciata* species, which corresponds to the species with the greatest mass in our study. This result was expected, given the tendency for increased MP in larger organisms due to higher food consumption^37^.

In contrast, when comparing total MP contamination levels weighted by mass to mitigate the effect of size variation between species, the highest average was found in *P. ephippifer*. Additionally, a higher level of MP contamination for this species was observed in all separately analyzed systems when also weighted by total mass. In fish, when analyzing different species considering size variations, a similar result was observed when standardizing contamination levels by total mass, with larger species showing a lower quantity of MP^37^. Therefore, although larger species are expected to bioaccumulate more MPs, processes such as the dilution of contamination can lead to a reduction in bioaccumulation indices when contamination levels are weighted by body mass.

In a recent study on adult anuran species, it was observed that the terrestrial species *(Duttaphrynus himalayanus* = 0.75 MP/g) exhibited a higher level of MP exposure compared to others^29^. This result is similar to ours, as the species *Physalaemus ephippifer* has a terrestrial habit, while *Boana multifasciata* has an arboreal habit. Thus, it is important to consider habitat variation among species, corresponding to different microhabitat uses^30^. The forest floor environment, with reduced surface runoff and higher soil permeability, contributes to this phenomenon, acting as a temporary or permanent sink through physical and biological processes^38,39^. This could justify the higher incidence of MPs observed in *Physalaemus ephippifer*. On the other hand, for arboreal species like *Boana multifasciat*a, airborne transport may represent the primary mechanism of MP exposure^32^. Additionally, the airborne translocation of microplastics promotes the adhesion of these particles to leaf surfaces and other plant structures, which can slow down the plastic degradation process^40^. However, although airborne transport and deposition on leaf structures significantly contribute to MP exposure levels, contamination levels in the soil tend to be higher, which aligns with our findings^39^. Additionally, studies addressing MP contamination in arboreal species remain scarce, making this the first record of MP contamination in adult arboreal anurans.

When comparing the levels of MP contamination in *P. ephippifer* and *B. multifasciata* with previous studies on adult anuran amphibians, we can only utilize the records from the gastrointestinal tract (GIT). For *P. ephippifer*, the mean MP per individual in the GIT was 1.42 ± 1.72, and for *B. multifasciata*, it was 2.13 ± 2.11 MP per individual. These values are similar to those obtained in a survey conducted in a forested area away from urban centers, with a frequency of 1 MP per individual^10^. In another study assessing contamination in adult anuran amphibians along an urbanization gradient, our averages resemble those in areas with lower anthropogenic influence (2.40 MP per individual)^11^. However, these values differ significantly from previous studies conducted in heavily anthropized areas. For *Pelophylax* spp. species, an average of 18.93 MP per individual was obtained in one study^11^, and another study reported an average of 18.60 MP per individual^29^, both in areas heavily influenced by human activity. This supports the hypothesis that individual contamination levels primarily reflect microhabitat contamination levels.

The proximity to large urban centers promotes a higher degree of MP contamination due to being major sources of improperly discarded plastic waste, in addition to industrial and agricultural activities releasing a significant amount of plastic waste into the environment^41,42^. However, the study area in the present work is not characterized by high urbanization or intensive industrial and agricultural activities nearby. There are low-density human occupations, and the area is commonly visited for ecotourism and environmental education activities. While populations with lower density have the capacity to pollute intensely with plastic waste^43^, in this context, it is valid to consider that the presence of MP does not depend solely on local contamination^42,44^. It can also be influenced by the transport from more distant areas, which can occur through abiotic transport (atmospheric and leaching) or biotic transport (trophic transfer of species with larger home ranges)^44^. Furthermore, given the dual life cycle in anurans and the potential for MP bioaccumulation, exposure during the larval stage may influence the presence of MP during the adult phase^45^.

Contamination in GIT of adult anurans is already well-known for the group, as mentioned earlier. However, we observed that the studied model species exhibited different responses when analyzing the effect of GIT morphometric measures on the level of MP contamination. The relationship observed between GIT morphometric measures and the level of MP contamination in the species *B. multifasciata* was negative. In an experimental study conducted with tadpoles, it was demonstrated that, in the long term, individuals with higher food consumption may have more efficient excretion for MP release, resulting in lower accumulation in the GIT^20^. This does not exclude potential effects on organisms^19,20^. This mechanism could explain the observed relationship between mass and the level of MP in the GIT of *B. multifasciata*. Additionally, the dilution of contamination due to an increase in organ length should be considered, as MP variability in the GIT was lower for *B. multifasciata*. In the case of *P. ephippifer*, the greater mass, which may represent higher food consumption, resulted in greater MP ingestion, reflecting a more contaminated environment and a higher level of contamination through trophic transfer^37^.

The mechanisms of trophic transfer of MP are primarily influenced by variations in diet among species^46^. For the species *P. ephippifer*, during the adult phase, the diet is predominantly composed of ants and termites^47^. In contrast, there is no specific dietary description for the *B. multifasciata* species in the literature; however, for the genus, a predominance in the consumption of grasshoppers, beetles, and hemipterans can be observed^48^. Although assessments of MP in terrestrial insects are still scarce, it is known that the contamination level varies among insect species, and their contamination also reflects the contamination level of the microhabitat to which they are exposed^49,50^. From this, it can be inferred that the main food sources of the model species vary in terms of microhabitat use and vary in terms of dispersal rate and home range.

Regarding dermal exposure, being an organ with direct contact with the environment, the amount of microplastics (MP) was found to be elevated^51^, and this exposure can occur through contact with contaminated areas (soil, water, and vegetation) or deposition (atmospheric precipitation) of microplastic particles^52^. The amphibian integument is a complex multifunctional organ, including functions related to respiration, defense, and immunity^53,54^. Although the effects of microplastics on the integument in anurans are not well-known, it is understood that they have highly permeable skin, increasing vulnerability (RT), this can be related especially to the inhalation of airborne particles, which can be local or transported from more distant locations^55^. This exposure pathway showed a lower quantity of plastic particles, like observations when comparing different days of exposure to toxic metals, where the risk of exposure is higher through ingestion, followed by dermal and inhalation pathways^51^, even in plastic particles deposited on the skin, not embedded in the tissue.

The discussion on the mechanisms and effects of MP inhalation has only recently emerged, with research primarily focused on humans^55,56^. There is no description of this type of contamination in terrestrial wildlife. From this, it is known that aerodynamic properties can influence deposition, with smaller particles being phagocytized by macrophages, while larger particles, especially fibers, which are predominant, may translocate to epithelial layers and accumulate^24,56^. However, although our data provide the first evidence that anurans are susceptible to MP inhalation, the effects of MP inhalation in adult anurans or tadpoles remain unknown.

Regarding the analysis of microplastic (MP) morphotypes, the predominance of the fiber shape was observed for both species and in all studied exposure pathways. The prevalence of the fiber shape, as well as the transparent color, was like the findings in a previous study on frogs of the genus *Pelophylax*, evaluating the spatiotemporal distribution in populations of this group in Turkey^36^ and in anuran populations in Bangladesh^29^. Microplastic fibers commonly originate from textile products and by-products and are often of secondary origin^42,57^. These fibers are characterized by being easily displaced between different natural compartments through airborne^23,58^ and aquatic transport^58,59^. Activities such as washing and drying synthetic fiber clothing can enhance the release of these particles into the environment^57^ and in the studied area, washing clothes in small water bodies is common. Additionally, the presence of people wearing synthetic clothing can release microplastic fibers into the environment. In addition to transparent, blue colors have been identified as predominant in previous studies on adult anuran amphibians^11,60^.

The polymers identified in this study have previously been documented in a study involving adult frogs^11^. Such polymers are widely used in a variety of industrial and consumer applications due to their physical and chemical properties. Polystyrene is a resilient polymer with high persistence, prevalent in aquatic environments^61^, commonly utilized in packaging, disposable containers, and thermal insulation. On the other hand, PET is renowned for its mechanical strength and low gas permeability^62^, making it an excellent material for manufacturing soda bottles and food packaging. Polyester, in turn, stands out for its durability and wrinkle resistance, employed in a range of products from clothing and textiles to engineering applications such as tire fibers and reinforced composites^63^. Given that the study area is not proximate to industrial zones, it is probable that these particles stem from improper waste disposal by surrounding residents, thus, linked to residential waste disposal.

In the context of the Amazon, research on microplastic (MP) contamination in the abiotic environment focuses on continental aquatic environments (rivers and lakes)^64,65^ and the coastal region^66^. Gerolin et al. (2020) identified the presence of microplastics in sediment in three major Amazonian rivers, with concentrations ranging from 417 to 8178 particles/kg of dry sediment, with the fiber morphotype being predominant. However, there is no research examining MP contamination in the soil of Amazonian Forest areas. This is the first study to indicate environmental contamination by MP in the terrestrial environment in the region based on evidence of contamination in bioindicator organisms.

From the analyses conducted in this study, it can be concluded that the species *Boana multifasciata* and *Physalaemus ephippifer* are exposed to MP contamination through all three exposure pathways: respiratory, dermal, and digestive. Additionally, we observed that total morphometric measures did not influence the total MP levels in the two species, while morphometric measures of the GIT influenced contamination levels in the GIT, which varied between species and may be related to the level of exposure through trophic transfer and food availability. Furthermore, variations in contamination indices between the model species indicated that *Physalaemus ephippifer* had a higher risk of exposure. Additionally, we identified that the digestive and dermal pathways showed higher levels of MP contamination, while the respiratory pathway was the least representative.

## Methods

### Study area and sampling protocol

The collections were carried out in the Gunma Ecological Park (PEG), located in the municipality of Santa Bárbara, Pará, Brazil. The PEG is situated in the Amazon Plain, covering an approximate area of 580 ha, with a predominant vegetation of dense ombrophiles forest on firm ground, areas of capoeira (secondary forests), floodplains, and igapós, featuring a humid tropical climate^67^.

The collections took place between January 2022 and April 2023, during the rainy season, corresponding to the reproductive period of the model species. Thirty adult individuals of each species, regardless of gender, were captured. The captures were performed through active search and manual collection. After capture, the animals were placed in previously sanitized glass containers and sent to the Multidisciplinary Laboratory of Animal Morphophysiology at the Federal University of Pará.

### Extraction and analysis of Microplastics (MPs)

The collected individuals were weighed to obtain total mass (g), and the measurement of snout-vent length (mm) (SVL) was recorded, using balance (0.001 g precision) and electronic caliper (0.05 mm precision). Subsequently, the individuals were euthanized using topical Lidocaine 0.2%. With the euthanized individuals, we performed dissection to obtain the gastrointestinal tract (GIT) (stomach and intestines), respiratory tract (RT) (trachea and lungs), and dorsal integument (Integ) (dorsal region of the abdomen, from pelvic to shoulder girdle), which were identified and stored separately in 5 ml glass containers. For the Integ, the particles deposited on the tissue and inserted into the tissue were considered. Next, the biometrics of the GIT were conducted, measuring the organ’s mass (mg) and length (mm).

Following the dissection of samples, digestion was carried out using 10% Potassium Hydroxide (KOH) (previously filtered with a cellulose acetate filter with a porosity of 0.2 μm). The RT samples remained in the digestion solution for 24 h, while the GIT and Integ samples stayed for a minimum of 48 h, or until the digestion was completed within a maximum of 96 h. All samples were kept in an oven at 50 °C for at least 24 h. The digested samples were filtered using ME24 cellulose acetate filters with a porosity of 0.2 μm, which were then placed in a glass Petri dish for subsequent analysis^68^.

### Identification and analysis of microplastic

For the visual analysis of microplastics, each filter was examined by scanning its entire length with the aid of a stereomicroscope at a magnification of 100x Zeiss Discovery V20 (Zeiss, Germany) equipped with *xyz* controlling software to obtain images and morphometric measurements. From this, the shape (fiber, granule, and fragment) and color (transparent, blue, red, and black) were determined, following the protocol recommended by Hu et al. (2022).

For the chemical characterization and validation of our samples, we used the Raman spectroscopy method^69^. To make the Raman measurements, the equipment LabRAM HR Evolution (HORIBA) was used, which has four different lasers to excite the sample (473 nm, 532 nm, 633 nm e 785 nm) and a 50x long range objective (NA = 0.55). The resistance of each material for different power levels for each laser was tested, to obtain the best signal without damaging the material. The spectral region between 200 and 3200 cm^− 1^ was used, which is typical for polymer studies^70^.

Different parameters were optimized along the measurement, particularly the laser wavelength and the maximum power that each sample could handle. To further improve the signal, the integration time, accumulation numbers or the diameter of the pinhole were shifted to get the best signal/noise ratio. A filter was used to automatically remove spike dues to cosmic rays, and through a MatLab code a baseline was applied to remove the background from the measurements. The spectra were identified using the KnowItAll^®^ program.

### Quality Assurance and Quality Control (QA/QC)

To reduce the contamination rate during handling, protective measures were adopted during sample collection, transportation, processing, and analysis. Items made of plastic were avoided at all stages, with a preference for glass and aluminum. Pre-filtered distilled water was used for equipment cleaning and preparation of solutions. The sample dissection was carried out in a fume hood with an exhaust system. Containers containing digested samples were opened only during the filtration stage. Both during digestion and after filtration, samples were shielded from light using aluminum foil. Circulation of people was restricted during dissection, filtration, and visual analysis, and a procedural blank sample was obtained^70,71^. The laboratory plastic contamination load was subtracted from the quantification of identified MPs in the blank samples. Due to the potential presence of plastic fibers in clothing, 100% cotton clothes and lab coats were used.

To ensure the quality of identified MP samples, inclusion criteria proposed by Ribeiro-Brasil (2020) were followed. Additionally, a hot needle test was conducted on suspicious particles, as the hot point makes plastic sticky and leaves a mark^72^. All identified samples were compared (in terms of morphotype and coloration) with previous studies that confirmed the polymer type^11,29,45^.

### Data analysis

Initially, we determined the composition of microplastics (MPs) in terms of shape (fiber, fragment, and granule) and color (blue, black, transparent, and red) based on the percentage of the total quantity of identified MPs for each species. To check for significant differences in MP sizes according to the exposure pathway, we subjected the data to the non-parametric Kruskal-Wallis test. To determine the effect of total mass and SVL on the total MP load and the effect of mass and length of the gastrointestinal tract (GIT) on GIT contamination, a Generalized Linear Model (GLM) was performed for each species. In this procedure, non-collinear morphometric measurements were considered as predictor variables, and the quantity of MPs was considered as the response variable, using the Poisson distribution family.

As contamination indices were determined by the ratios: (i) the total number of MPs per individual (MP/ind), (ii) total number of MPs per gram (MP/g), (iii) total number of MPs per SVL (MP/SVL), (iv) and number of MPs in the GIT per mass of the GIT (MP GIT/mass GIT). The variation in MP contamination indices between the two species and according to different exposure pathways was tested using Permutational Multivariate Analysis of Variance using Distance Matrices (PERMANOVA)^73^ in conjunction with Multivariate Homogeneity of Groups Dispersions (PERMDISP) and we used the Bray-Curtis dissimilarity matrix^74^, both performed with 9999 unrestricted permutations. For the evaluation of multiple comparisons, we used the “pairwise.adonis” function implemented in the *pairwiseAdonis* package, with p-values adjusted by the Bonferroni method. We also used this procedure (PERMANOVA and PERMDISP) to analyze the variation in MP contamination considering systems and species as independent predictor variables and the number of MPs weighted and unweighted by total mass as the response variable. PERMANOVA was conducted using the adonis2 function, and PERMDISP was performed using the betadisper function, both implemented in the *Vegan* package. All analyses were carried out in the R environment (R 4.3.1)^75^.

## Data Availability

The data on which this article is based are available at the following link: https://doi.org/10.6084/m9.figshare.26064448.v1.
